# Function scores of different surgeries in the treatment of knee osteoarthritis: A PRISMA-compliant systematic review and network-meta analysis

**DOI:** 10.1097/MD.0000000000010828

**Published:** 2018-05-25

**Authors:** Cheng-Yao Liu, Chuan-Dong Li, Liang Wang, Shan Ren, Fu-Bin Yu, Jin-Guang Li, Jiang-Xiong Ma, Xing-Long Ma

**Affiliations:** a Department of Bone and Joint Surgery; b Department of Bone Trauma, The Sixth People's Hospital of Ji’nan City, Ji’nan, PR China.

**Keywords:** cohort study, efficacy, function score, knee osteoarthritis, network-meta analysis, surgical treatment

## Abstract

**Background:**

Osteoarthritis (OA) is the third most common diagnosis made by general practitioners in older patients. The aim of this study was to compare the function scores of different surgeries in the treatment of knee osteoarthritis (KOA).

**Methods:**

Cohort studies about different surgical treatments for KOA were included with a comprehensive search in PubMed, Cochrane Library, and Embase. The standard mean difference (SMD) value was evaluated and the surface under the cumulative ranking (SUCRA) curve was drawn with a combination of direct and indirect evidence. A total of 265 eligible patients were enrolled and served as the nonoperative treatment group, osteotomy group, unicompartmental knee arthroplasty (UKA) group, total knee arthroplasty (TKA) group, and arthroscopic surgery group. Before surgery, 6 months after surgery, 1 year after surgery and 5 years after surgery, the hospital for special surgery (HSS) knee score, Lysholm score, Western Ontario and McMaster Universities Osteoarthritis Index (WOMAC) score, and American knee society score (KSS) were recorded.

**Results:**

A total of 9 cohort studies including 954 patients with KOA were finally enrolled into the study. The network-meta analysis revealed that osteotomy and UKA treatments showed a better efficacy on improving the function score. Our cohort study further confirmed that, a higher HSS knee score after 1 year and higher Lysholm score after 6 months and 1 year were observed in the osteotomy and UKA groups, while better HSS knee score and KSS after 6 months and 1 year were showed in the osteotomy and TKA groups. In the TKA group, Lysholm score and KSS were higher and WOMAC score was lower after 5 years than other groups. WOMAC score was lowest in the UKA group after 6 months, 1 year and 5 years of surgery.

**Conclusion:**

These results provide evidence that function scores of patients with KOA were improved by osteotomy, UKA, TKA, and arthroscopic surgery. And osteotomy and UKA showed better short-term efficacy, while TKA appeared better long-term efficacy.

## Abstract 2

Supplemental Digital Content is available in the text

## 1 Introduction

Knee osteoarthritis (KOA) is a progressive disease involving the intra-articular tibiofemoral and patellofemoral cartilage.^[1]^ It may affect any joint within the body causing chronic pain, functional limitation, and emotional disturbance and may lead to disability and negatively affect quality of life.^[2,3]^ As a common phenomenon in elderly, KOA affecting approximately 3.64% of people around the world in 2010.^[4]^ For KOA treatment, many kinds of regimens have been constructed, including nonpharmacological (exercise and changes of lifestyle) and pharmacological methods (analgesics and corticosteroid injections), and the first one often accompanied with some side effects.^[5,6]^ Furthermore, in addition to the above mentioned pharmacological and nonpharmaceutical interventions, there are more expensive surgical interventions, often limited to patients who made no respond to other treatments.^[7]^

As the number of older person increases, the need for surgical treatment, such as knee arthroplasty, increases thereby which accompanied with increased costs in treating KOA.^[8]^ Statistic demonstrated that osteotomy is a widely accepted treatment method for medial compartment KOA, especially for those young and active patients,^[9]^ but its procedures have been ignored by most surgeons due to the technological improvements and the early term success of the resurfacing and unicompartmental knee arthroplasty (UKA) procedures.^[10]^ UKA, first introduced in the 1970s is known as a joint resurfacing method in which affected degenerative chambers were implanted with prostheses and unaffected intervals were preserved.^[9]^ Total knee arthroplasty (TKA) is the most commonly performed joint replacement surgery worldwide and its major beneficial effects are pain relief, increases range of movements, and better quality of life.^[11]^ In the USA, arthroscopic surgery is the most common orthopedic procedure for patients suffering KOA, but little is known about patients’ expectations concerning recovery time and leisure-time activities after surgery.^[12]^

These 4 surgical treatments have their own advantages and disadvantages, but the studies that compare the outcomes of these 4 surgical treatments and their effects are lacking.^[9]^ Furthermore, there was a previous study hypothesized that there would be no significant difference in the cost-effectiveness or functional outcomes of the different surgical treatment options for KOA.^[13]^ In order to compare which surgery was the optimal treatment for KOA, we compare the functional outcomes such as hospital for special surgery (HSS) knee score, Lysholm score, Western Ontario and McMaster Universities Osteoarthritis Index (WOMAC) score, and American knee society score (KSS) of different surgical treatments for KOA using a NMA and cohort study, expecting this study will be helpful for patients to choose the best surgical treatment for KOA.

## 2 Materials and methods

### 2.1 Search strategy

Relevant cohort studies were searched by computer-based retrieval from PubMed, Cochrane Library, and Embase electronic databases (from the inception to May 2017) combined with manual retrieval. The search strategy of key words combined with free words was conducted with following search terms: surgical treatment; osteotomy; unicompartmental knee arthroplasty; total knee arthroplasty or total knee replacement; arthroscopic surgery; knee osteoarthritis; cohort study, etc.

### 2.2 Inclusion and exclusion criteria

The inclusion criteria were as follows: study design should be cohort study; surgical treatments included nonoperative treatment; osteotomy, UKA, TKA, and arthroscopic surgery; study subjects should be patients with KOA; outcomes included knee injury and osteoarthritis outcome score (KOOS), Lysholm score, Western Ontario and McMaster Universities Osteoarthritis Index (WOMAC) score, hospital for special surgery (HSS) knee score, American knee society knee score (KSS), and British Orthopaedic Association (BOA) score. Lysholm is a conditional specificity score for evaluating knee ligament injury. It can not only evaluate the functional perception of patients’ daily activities, but also make a preliminary assessment of patients’ different intensity of motor function grades. HSS score, brought up by American hospital for special surgery in 1976, is a scoring system in total of 100 points, can only be used to compare functional recovery before and after operation in patients, and cannot assess the risk of operation correctly, so the usage decreased gradually. WOMAC score was first proposed by Bellamy et al in 1988. This score is based on the relevant symptoms and signs of patients to evaluate the severity of KOA and the therapeutic effect, which can reflect the degree of inflammation of patients. KOOS was developed from the earliest WOMAC, and it is more comprehensive and widely used. KSS score is to obtain joint anatomy, biomechanics, and other information, and to understand the functional recovery of patients through the examiner interview and physical examination. BOA score employs the revised BOA knee function assessment chart with a total of 39 points, including 4 points for pain, 17 for function, and 18 for malformation, the higher the scores are, the better the effect is. The exclusion criteria were as follows: patients treated with previous surgery or joint arthroplasties on the same knee within the past six months; patients with history of inflammatory or septic arthritis, or patients with fracture; studies with incomplete literature data (e.g., nonmatched pair studies); studies were noncohort studies, conference reports, systematic reviews and abstracts, non-English studies, nonhuman studies, and duplicate studies.

### 2.3 Data extraction and quality assessment

With the standard data collection forms, data from included studies was extracted by two researchers independently. Any disagreements were resolved through discussion. The quality of included studies was assessed using the Newcastle–Ottawa scale (NOS) assessments, which including 10 items: representativeness of the exposed cohort, selection of the nonexposed cohort, ascertainment of exposure, demonstration that outcome of interest was not present at start of study, comparability, assessment of outcome, was follow-up long enough for outcomes to occur, adequacy of follow-up of cohorts, total NOS score, and total categorized NOS score.^[14]^ As the total points were 9 points, studies with > 5 points were included in this NMA.

### 2.4 Study subjects

Between October 2010 and October 2011, a total of 265 patients with KOA with complete clinical data were selected from the Sixth People's Hospital of Ji’nan City. All enrolled patients were diagnosed with KOA according to the diagnostic criteria^[15]^ and were received different surgical treatments for the first time. Based on surgical treatments, patients were assigned into 5 groups: the nonsurgical treatment group (as a control, n = 48), the osteotomy group (n = 53), the UKA group (n = 59), the TKA group (n = 62), and the arthroscopic surgery group (n = 43). The inclusion criteria were: patients with persistent knee pain over 3 months; patients were diagnosed with KOA using nuclear magnetic resonance examination; patients were treated with surgical treatments for KOA for the first time; patients aged > 20 years old with complete follow-up data; patients were conscious of receiving the prescribed treatments and were willing to sign the written informed consent. The exclusion criteria were: patients were enrolled for no more than 6 months before surgery; patients’ diagnosis were not supported by imaging examinations; patients with mental illness, cardiopulmonary compensatory function, serious neoplastic diseases, or other organs of severe failure; patients with pregnancy or lactation; patients refused to sign the informed consent. The protocol of this study was carried out with the approval of the ethics committee of the Sixth People's Hospital of Ji’nan City. All enrolled individuals and their families signed the written informed consent. All the study procedures were in line with the Declaration of Helsinki.^[16]^

### 2.5 Surgical procedures

All patients in the 4 surgical treatment groups were supine and received nerve blocking anesthesia. The osteotomy group: fibular osteotomy was performed at one third of fibula and 1 cm of fibula was cut off. After cut a transverse incision at the upper border of tibial tubercle, the detachment of the periosteum of lateral and posterior aspects of tibia was performed under tibial plateau. Then the periosteal detacher (Shanghai LZQ Precision Tool Technology Co., Ltd., Shanghai, China) was placed under the periosteum to protect the nerves and blood vessels of the posterior aspect of the knee. Two injection needles were inserted into the extra-articular and intra-articular space of the knee to determine the surface of tibial plateau. At a distance of 2 cm from the surface of tibial plateau and at the parallel to the articular surface, the sphenoid bone was cut from inside to outside. With extension and extroversion of the knee, the medial cortex was incompletely amputated, and the cross-section of leg was closed. Tibial tubercle was elevated and distal tibia was moved forward by 1 to 1.5 cm. The UKA group: after separated the subcutaneous soft tissues and pulled the patella to the opposite side, the anterior cruciate ligament, meniscus and the partial infrapatellar fat pad were removed and intra-articular injuries were cleaned up. In order to measure the balancing of the flexion-extension gap, bone tissues proliferated at the margin of the medial aspect of tibial were excised, tibial osteotomy and femoral osteotomy were carried out and femur models (Shanghai LZQ Precision Tool Technology Co., Ltd., Shanghai, China) were installed. After the measurement, unicompartmental knee prosthesis was implanted and the knee was sutured. The TKA group: the skin, subcutaneous tissues, and capsular ligament were cut in sequence, and the patella was extroverted after an incision was cut at the medial aspect of the knee. The articular surface of the tibia was removed, soft tissues with internal and external contractures were released and then the osteophyte was removed. The restoration of the axial alignment of the lower extremity was performed and the patella was not replaced. The arthroscopic surgery group: after diagnostic arthroscopy, torn and degenerative meniscus, the fragments of cruciate ligaments and the hyperplastic synovium were removed under arthroscope, while the fragments of articular cartilage and the loose bodies in the knee were removed. Patients in the nonsurgical treatment group were not received surgical treatments but treated with the treatment combined with traditional Chinese and Western medicine. Diacerein, an inhibitor of interleukin-1 (IL-1), was taken 100 mg during 0.5 to 1 hour after the meal, and meloxicam capsules 7.5 mg were administered 3 times daily. Meanwhile, the traditional Chinese medicine that mainly promoting blood circulation, dredging collateral, and nourishing the liver and kidney were taken. The prescription including 15 g of Eucommia ulmoides, 10 g of Acanthopanacis cortex, 15 g of Lycopodium clavatum, 15 g of Loranthus parasiticus, 15 g of Speranskia tuberculata, 15 g of Liquorice, 15 g of Achyranthes bidentata, and 15 g of Radix clematidis, and patients were reviewed regularly.

### 2.6 Observation and evaluation

HSS scores,^[17]^ Lysholm scores,^[18]^ WOMAC scores^[19]^ and KSS scores^[20]^ of patients were recorded before surgery, 6 months after surgery, 1 year after surgery and 5 years after surgery.

*HSS score*: totally 100 points, including pain (30 points), function (22 points) and activity (18 points) as well as flexion deformity, myodynamia, and stability (10 points respectively). Penalized items included: patients with a walking stick or a single crutch each minus 1 point; patients’ knee with 15° extension lag minus 5 points; patients’ knee at 5° varus or 5° valgus minus 1 point. Scores > 85 points were excellent, 70 to 84 points were good, 60 to 69 points were qualified, and <60 points were bad.

*Lysholm score*: totally 100 points, included: claudication (mild intermittent claudication/severe persistent claudication) and weight each accounted for 5 points; whether there was a locking knee and the frequency accounted for 15 points; whether there was a joint instability during movement or heavy work, and whether there was a joint pain after work or walking more than 2 km each accounted for 25 points; whether there was a swelling after heavy work or normal activity, whether there was a difficulty during walking up or down the stairs and the degree of follow-up each accounted for 10 points; whether there was a difficulty during a deep knee bend or whether a deep knee bend exceeded 90° accounted for 5 points, respectively. Scores >87 points were excellent, 77 to 86 points were good, 66 to 76 points were qualified, and <66 points were bad.

*WOMAC score*: written by patients and only scored the lesions of the knee. Scores were recorded on physical function, pain and the degree of stiffness and divided into 5 grades: without (0 points), mild (1 point), moderate (2 points), severe (3 points), very severe (4 points).

*KSS score*: totally 200 points. Pain accounted for 50 points; the range of motion of the knee, the stability of anterior and posterior aspects of the knee, and the stability of lateral and medial aspects of the knee totally accounted for 50 points; whether there was a difficulty during walking or walking up or down the stairs accounted for 100 points.

### 2.7 Statistical analysis

Firstly, the fixed-effects model was used to perform pairwise meta-analyses of direct evidence with R version 3.2.1 and the Meta package. The pooled estimates of standard mean difference (SMD) value and its 95% credible intervals (CIs) of outcomes were measured by a fixed or random effect model. I-square test and Chi-square test were conducted to detect the heterogeneity among the enrolled studies.^[21]^ Secondly, the Meta package of R 3.2.1 software was applied to draw network meta diagram, in which each node represented different intervention, the node sizes reflected sample sizes, and the thickness of lines between nodes meant numbers of included studies. Thirdly, a random-effects NMA with the gemtc package was conducted. It models the relative effects (e.g., SMD fitting a generalized linear model (GLM) under the Bayesian framework by linking to JAGS, OpenBUGS or WinBUGS as first described by Lu and Ades^[22]^ and extended by others.^[23,24]^ To assist in the interpretation of SMDs, whether the probability of each intervention to be the most effective or safest treatment method was calculated according to a Bayesian approach by probability values which were drawn as the surface under the cumulative ranking (SUCRA) curves; and the rank of the intervention was better if the SUCRA value was larger^[25,26]^ SPSS 18.0 software (IBM Corp. Armonk, NY) was performed for the statistical analysis, with measurement data presented as mean ± standard deviation. Comparisons among multiple groups were done by one-way analysis of variance (ANOVA), and then comparisons between two groups were done by the least significant difference (LSD) test, while comparisons of different time points before and after surgery in a group were done by repeated measures ANOVA. *P* < .05 was accepted as indicative of statistical significance.

## 3 Results

### 3.1 Nine cohort studies are included in this NMA

**Figure 1:**
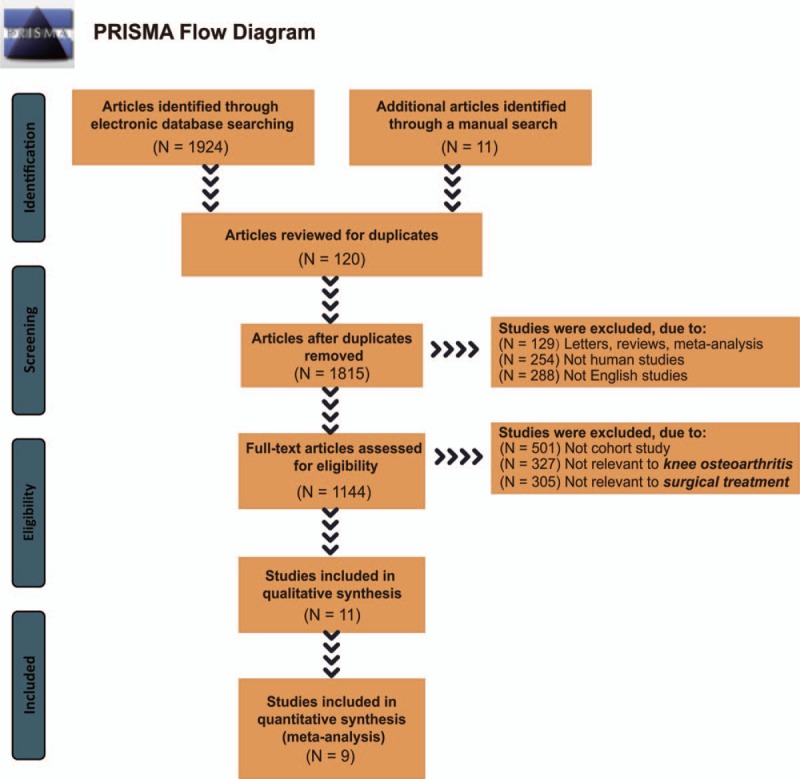
Flowchart for literature search selection. Note: Nine cohort studies that met the inclusion criteria were included in this network meta-analysis. N = numbers.

**Figure 2:**
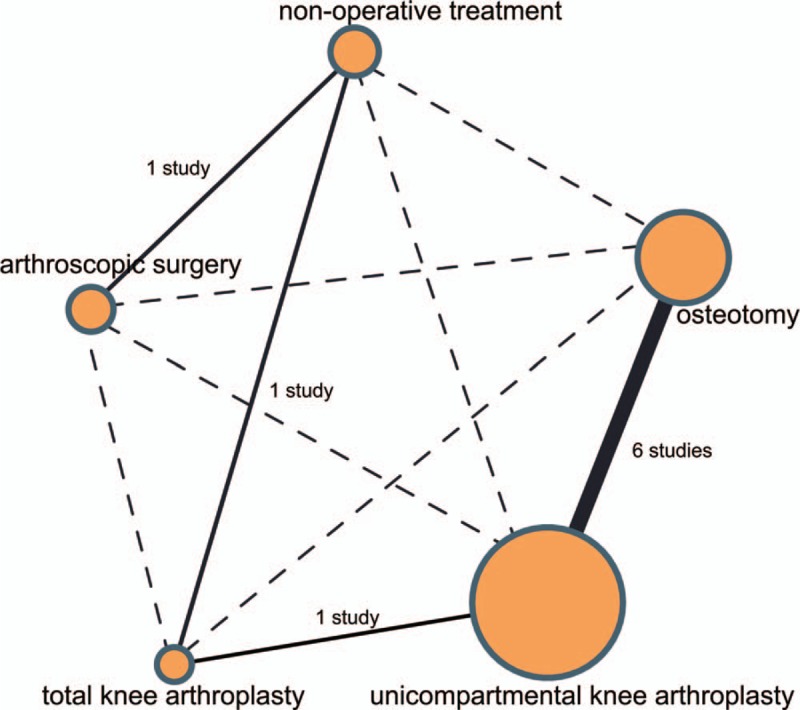
Network evidence of the comparisons for the efficacy of different surgical treatments for KOA. KOA = knee osteoarthritis.

A total of 1935 relevant studies were initially retrieved. We firstly excluded 120 duplicate studies, 129 letters or reviews, 254 nonhuman studies, and 288 non-English studies. After full-text review, the remaining 1144 studies were conducted with further exclusion, 501 noncohort studies, 327 unrelated to KOA, 305 unrelated to surgical treatments, and 2 without data integrity or with no data were ruled out. Finally, 9 cohort studies were eligible to this NMA^[19,27–34]^ (Fig. 1). These 9 cohort studies were published from 1998 to 2017 and all of them were two-arm trials. Individuals were aging from 40 to 90 years old, and subjects in 7 studies were Caucasians and in 2 studies were Asians. The baseline characteristics of these included studies are displayed in Supplementary Table 1. The NOS assessments results are shown in Supplementary Fig. 1. Studies focused on the comparisons between osteotomy and UKA are relatively larger while the sample sizes of UKA are larger (Fig. 2).

### 3.2 UKA had a better efficacy in the treatment of KOA

**Figure 3:**
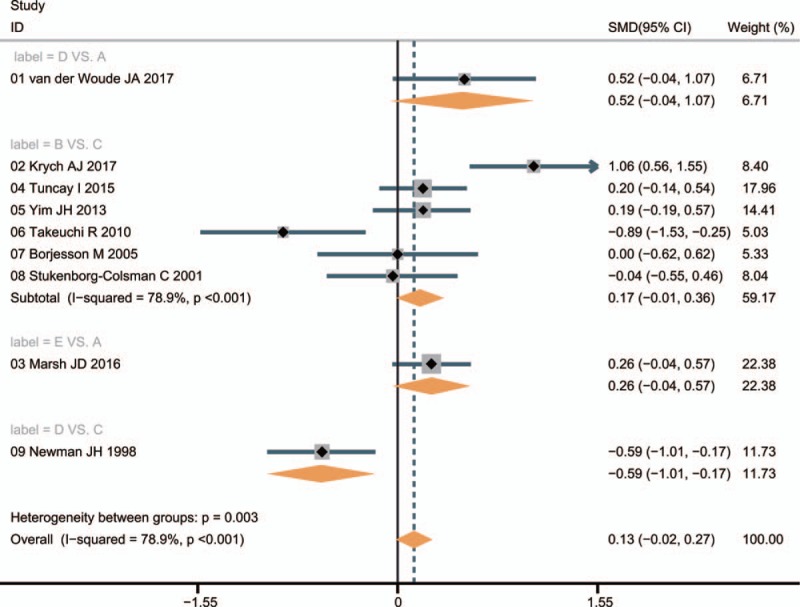
Forest plots of the comparisons for the efficacy of different surgical treatments for KOA. A = nonoperative treatment (control); B = osteotomy; C = unicompartmental knee arthroplasty; D = total knee arthroplasty; E = arthroscopic surgery; KOA = knee osteoarthritis.

Patients treated with UKA had a better improvement function scores than those treated with TKA (SMD = −0.59, 95%CI = −1.01 to −0.17); while compared with nonoperative treatment, there was no significant difference in the improvement of KOA in patients received TKA and arthroscopic surgery (SMD = 0.52, 95%CI = −0.04 –1.07; SMD = 0.26, 95%CI = −0.04–0.57, respectively). No markedly difference was observed in the improvement of KOA in patients with osteotomy and UKA treatments (SMD = 0.17, 95%CI = −0.01–0.36). These results indicated that UKA had a better efficacy in the treatment of patients with KOA (Fig. 3).

### 3.3 Osteotomy and UKA illustrated better treatment of KOA by proved by Network evidence

Results of indirect comparisons showed that there was no evidently difference in the efficacy of different treatments for KOA. Compared with nonoperative treatment, the other 4 operative treatments had a better improvement efficacy on function scores of patients with KOA, while osteotomy and UKA showed relatively better effects, followed by TKA, and arthroscopic surgery appeared relatively poor effect (Supplementary Table 2).

### 3.4 Osteotomy and UKA demonstrated a better efficacy in the treatment of KOA verified by Sucra values

**Figure 4:**
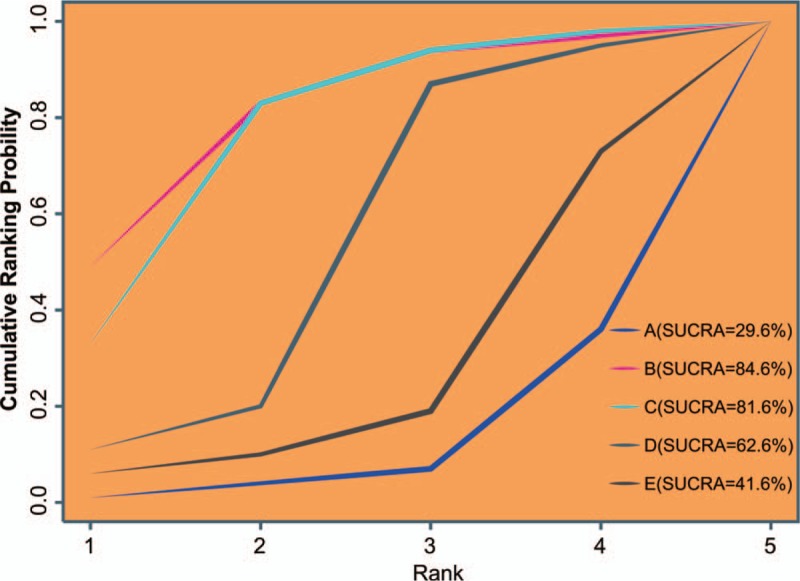
Cluster analyses for the efficacy of different surgical treatments for KOA. A = nonoperative treatment (control); B = osteotomy; C = unicompartmental knee arthroplasty; D = total knee arthroplasty; E = arthroscopic surgery; KOA = knee osteoarthritis.

As shown in Figure 4, the SUCRA values of nonoperative treatment, osteotomy, UKA, TKA, and arthroscopic surgery were 29.6%, 84.6%, 81.6%, 62.6% and 41.6%, respectively. These results revealed that patients treated with osteotomy and UKA had better function score improvement on KOA, which demonstrated that osteotomy and UKA had a better efficacy in the treatment of patients with KOA.

### 3.5 No publication bias assessment is found

**Figure 5:**
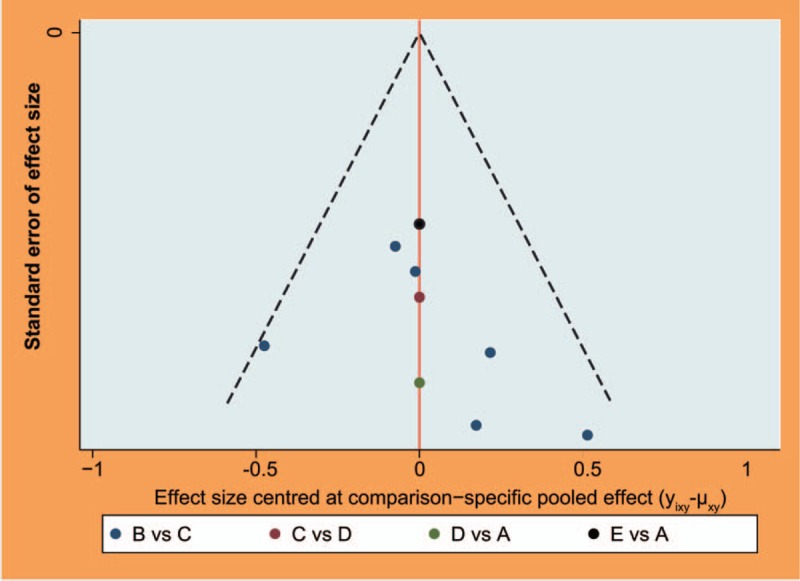
Funnel plots for the evaluation of publication bias of included studies. A = nonoperative treatment (control); B = osteotomy; C = unicompartmental knee arthroplasty; D = total knee arthroplasty; E = arthroscopic surgery; KOA = knee osteoarthritis.

Figure 5 shows that all scattered points are in the funnel and are of symmetric distributions at both ends of the red line, which reveals that there was no obviously publication bias.

### 3.6 All enrolled patients present comparable

As shown in Table 1, there was no evidently difference in age, gender, lesion location, course of disease, body mass index (BMI), diabetes mellitus, and hypertension among the nonoperative treatment, osteotomy, UKA, TKA, and arthroscopic surgery groups (*P* > .05), which indicated that the five groups were comparable.

**Table 1:**
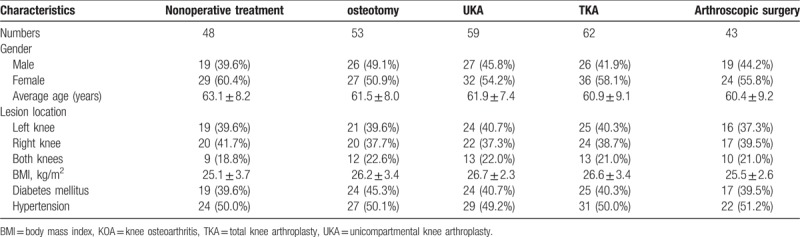
Baseline characteristics of patients with KOA in 5 groups.

### 3.7 Patients receive the osteotomy or UKA operations have the highest HSS scores

No marked difference was found in HSS scores of patients in the 5 groups before surgery (*P* > 0.05), but the HSS scores of patients at 6 months, 1 year, and 5 years after surgery in the osteotomy, UKA, TKA, and arthroscopic surgery groups were higher than that in the nonoperative treatment group (*P* < .05). Patients in the osteotomy and UKA groups had the highest HSS scores at 6 months and 1 year after surgery (*P* < .05), followed by the TKA group, and then the arthroscopic surgery group. The HSS scores of patients in the osteotomy and TKA groups were the highest at 5 years after surgery (*P* < .05), followed by the UKA group, and then the arthroscopic surgery group (Table 2).

**Table 2:**
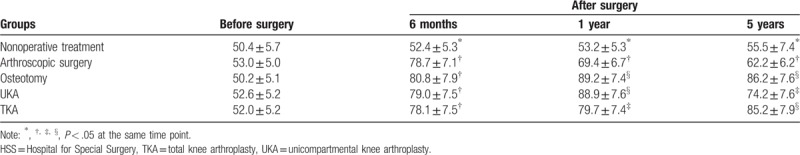
HSS scores of patients in five groups at different time points before and after surgery.

### 3.8 Patients receive the osteotomy or UKA operations have the highest Lysholm scores

These results are shown in Table 3. There was no obvious difference in Lysholm scores of patients before surgery among the five groups, but the Lysholm scores in the osteotomy, UKA, TKA and arthroscopic surgery groups were higher at 6 months, 1 year and 5 years after surgery than the nonoperative treatment group. Patients in the osteotomy and UKA groups had the highest Lysholm scores at 6 months and 1 year after surgery, followed by the TKA group; while the arthroscopic surgery group had lowest. The Lysholm scores at 5 years after surgery in the TKA group were the highest, followed by the osteotomy and UKA groups, and then the arthroscopic surgery group.

**Table 3:**
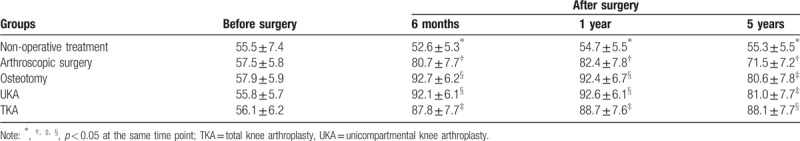
Lysholm scores of patients in five groups at different time points before and after surgery.

### 3.9 Patients receive UKA and TKA treatments have the lowest WOMAC scores

As shown in Table 4, no significant difference was observed in WOMAC scores of patients among the 5 groups before surgery, but the WOMAC scores at 6 months, 1 year, and 5 years after surgery were higher in the osteotomy, UKA, TKA, and arthroscopic surgery groups than the nonoperative treatment group. The UKA group had lowest WOMAC scores at 6 months and 1 year after surgery, followed by the osteotomy and TKA groups; while the arthroscopic surgery group had the highest. Patients in the UKA and TKA groups had the lowest WOMAC scores at 5 years after surgery, followed by the osteotomy group; while the arthroscopic surgery group had the highest WOMAC scores.

**Table 4:**
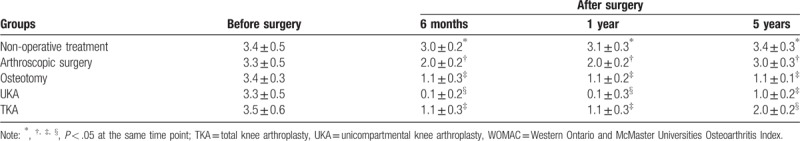
WOMAC scores of patients in five groups at different time points before and after surgery.

### 3.10 Patients receive osteotomy and TKA treatments have the highest KSS scores

The results shown in Table 5 suggested that there was no obviously difference in KSS scores of patients in the nonoperative treatment, osteotomy, UKA, TKA, and arthroscopic surgery groups before surgery, but the KSS scores of patients in the osteotomy, UKA, TKA, and arthroscopic surgery groups were higher at 6 months, 1 year, and 5 years after surgery than the nonoperative treatment group. Patients at 6 months and 1 year after surgery in the osteotomy and TKA groups had the highest KSS scores, followed by the UKA group, and then the arthroscopic surgery group. The KSS scores of patients in the TKA group were the highest at 5 years after surgery, followed by the osteotomy and UKA groups, and then the arthroscopic surgery group.

**Table 5:**
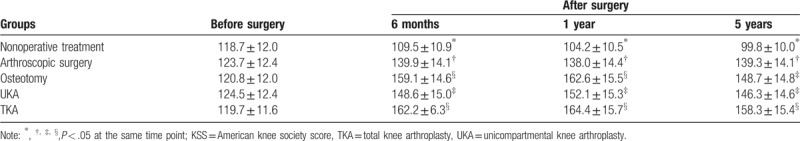
KSS of patients in five groups at different time points before and after surgery.

## 4 Discussion

In the present study, 9 cohort studies about 4 surgical treatments (osteotomy, UKA, TKA, and arthroscopic surgery) in the treatment of patients with KOA were enrolled in, and then a cohort study was conducted to further confirm the results. We come to the conclusion that osteotomy, UKA, TKA, and arthroscopic surgery could improve the function score of patients with KOA, while osteotomy and UKA had a better short-term efficacy, and TKA had a better long-term efficacy.

Our study demonstrated that KOA patients treated with osteotomy and UKA had a better function score improvement after 6 months or 1 year surgery than the other surgical treatments, which indicate that osteotomy and UKA may have a better short-term efficacy in the treatment of patients with KOA. Osteotomy had a positive effect on spinal alignment, and lower extremity alignment, as well as reduced the abnormality that may result in spinal problems such as degeneration or pain.^[35]^ KOA patients present a significant increase in the knee adduction moment and a medial shift in the dynamic knee loading, which will optimally restore cartilage loading forces and knee ligament balance and reduces progression or the risk of KOA.^[36]^ A study showed that osteotomy had a short-term efficacy and safety in patients with KOA.^[37]^ Besides, only at the first-year follow-up, all complications of the treatment were minor and the patients recovered without any problems.^[38]^ Patients’ knee function, postoperative pain, range of motion and deep vein thrombosis were getting better and the complication rate was less.^[39]^ Altuntas et al^[40]^ found that the short-term results of the domed tibia, mobile bearing lateral UKA supported the safety and efficacy of the procedure as a treatment option in the patients with KOA. There was a trend toward higher survival of prostheses for TKA than UKA in the follow-up between 3 and 10 years.^[39]^

Meanwhile, our study also demonstrated that KOA patients treated with TKA showed a better function score after 5-year surgery than other surgical treatments, which suggested that TKA may have a better long-term efficacy for KOA. Soft tissue balance is the most important surgical procedure for KOA, due to its great impact on the keen stability and mobility after the surgery.^[41]^ TKA exerts durability and effectiveness in the recovery of knee function.^[42]^ TKA could reduce the mechanical complications such as aseptic loosening and some other infections, at the same time, TKA also provided clinical improvement in knee function.^[43]^ In addition, no patellar fracture, joint instability or dislocation, vascular injury, common peroneal nerve injury, and deep vein thrombosis were observed following the TKA.^[44,45]^ The present study confirmed previous reports that TKA has a better long-term outcome in comparison with UKA.^[42]^ In addition, Bolognesi et al^[46]^ found that the 5-year revision rate was 3.7% for TKA and 8.0% for UKA.

Besides, the main results of NMA and cluster analysis revealed that the efficacy of arthroscopic surgery was relatively poor in the treatment of patients with KOA. Levels of keratan sulfate (KS), chondroitin 6-sulfate (C6S), synovial fluid biochemical markers, showed a strong correlation, and the levels of KS exhibited significant reduction, which indicates suppressed cartilage turnover after arthroscopic surgery.^[47]^ Physical function, pain, and health-related quality of life were not improved for patients with KOA even though they had been treated with arthroscopic surgery.^[48]^ KOA patients will suffer from frequent knee pain, cartilage damage, and degenerative meniscal tissue following arthroscopic surgery.^[49]^ Brignardello-Petersen R et al^[50]^ indicated that their results provided low-quality evidence that arthroscopic surgery is a safe procedure with a low risk of complications and moderated to high-quality evidence that the procedure provided very small benefits in pain and function over conservative therapy in the short term. Also, arthroscopic surgery provided no significant benefit over placebo surgery in patients with a degenerative meniscal tear and no KOA, and caution should be exercised for patients to choose arthroscopic surgery even after a failed attempt of conservative treatment.^[51]^

Although this study compared 4 functional outcomes such as hospital for special surgery (HSS) knee score, Lysholm score, Western Ontario and McMaster Universities Osteoarthritis Index (WOMAC) score, and American knee society score (KSS) of patients with KOA, it could not offer enough direct comparison of individual surgical treatment due to the limited references and data. Also, there are differences in the number of included studies under direct-paired comparisons between different surgical treatments and it may have effects on the results of our study. Despite these limitations, there are advantages to our research. The pair-wise meta-analysis and network meta-analysis were performed to comprehensively compare the function scores of patients treated with these four surgical treatments (osteotomy, UKA, TKA, and arthroscopic surgery) and the integration of existing evidence provides a referential direction for the clinical surgical treatment for KOA.

## 5 Conclusion

In conclusion, this study revealed that osteotomy, UKA, TKA, and arthroscopic surgery could improve the function scores of patients with KOA, among which osteotomy and UKA showed best short-term efficacy while TKA showed best long-term efficacy. Finally, we hope there will be more researchers to explore the efficacy and safety of different surgical treatments for patients with KOA, thus there will be more and more references and data to be referenced, which will provide a better theoretical basis for clinical treatment of KOA.

## Acknowledgments

We would like to acknowledge the helpful comments on this paper received from our reviewers.

## Author contributions

**Conceptualization:** Cheng-Yao Liu, Chuan-Dong Li, Liang Wang, Shan Ren, Fu-Bin Yu, Jin-Guang Li, Jiang-Xiong Ma, Xing-Long Ma.

**Data curation:** Cheng-Yao Liu, Chuan-Dong Li, Liang Wang, Shan Ren, Fu-Bin Yu, Jin-Guang Li, Jiang-Xiong Ma, Xing-Long Ma.

**Formal analysis:** Cheng-Yao Liu, Chuan-Dong Li, Liang Wang, Shan Ren, Fu-Bin Yu, Jin-Guang Li, Jiang-Xiong Ma, Xing-Long Ma.

**Funding acquisition:** Cheng-Yao Liu, Chuan-Dong Li, Liang Wang, Shan Ren, Fu-Bin Yu, Jin-Guang Li.

**Investigation:** Cheng-Yao Liu, Chuan-Dong Li, Liang Wang, Shan Ren, Fu-Bin Yu, Jin-Guang Li.

**Methodology:** Cheng-Yao Liu, Chuan-Dong Li, Liang Wang, Shan Ren, Fu-Bin Yu, Jin-Guang Li, Jiang-Xiong Ma.

**Project administration:** Cheng-Yao Liu, Chuan-Dong Li, Liang Wang, Shan Ren, Fu-Bin Yu, Jin-Guang Li.

**Resources:** Cheng-Yao Liu, Chuan-Dong Li, Liang Wang, Shan Ren, Fu-Bin Yu, Jin-Guang Li.

**Software:** Cheng-Yao Liu, Chuan-Dong Li, Liang Wang, Shan Ren, Fu-Bin Yu, Jin-Guang Li.

**Supervision:** Cheng-Yao Liu, Chuan-Dong Li, Liang Wang, Shan Ren, Fu-Bin Yu, Jin-Guang Li.

**Validation:** Cheng-Yao Liu, Chuan-Dong Li, Liang Wang, Shan Ren, Fu-Bin Yu, Jin-Guang Li.

**Visualization:** Cheng-Yao Liu, Chuan-Dong Li, Liang Wang, Shan Ren, Fu-Bin Yu, Jin-Guang Li.

**Writing – original draft:** Cheng-Yao Liu, Chuan-Dong Li, Liang Wang, Shan Ren, Fu-Bin Yu, Jin-Guang Li.

**Writing – review & editing:** Cheng-Yao Liu, Chuan-Dong Li, Liang Wang, Shan Ren, Fu-Bin Yu, Jin-Guang Li.

## Supplementary Material

Supplemental Digital Content
