# Supplementary material for: Function scores of different surgeries in the treatment of knee osteoarthritis: A PRISMA-compliant systematic review and network-meta analysis
Source: Medicine (Baltimore). 2018 May 25;97(21):e10828. doi: 10.1097/MD.0000000000010828 (PMC6393067; doi:10.1097/MD.0000000000010828)
Supplement: Supplemental Digital Content [file medi-97-e10828-s001.doc]

**Supplementary Fig. 1.** Quality assessment of included studies using NOS score. Note: NOS░=░Newcastle-Ottawa quality assessment scale.

**Supplementary Table 1.** Baseline characteristics of included studies

| First author | Year | Country | Ethnicity | Functional scores | Interventions | | Total | Sample size | | Age (years) | | M/F | |
| --- | --- | --- | --- | --- | --- | --- | --- | --- | --- | --- | --- | --- | --- |
|  |  |  |  |  | T1 | T2 |  | T1 | T2 | T1 | T2 | T1 | T2 |
| van der Woude JA | 2017 | Netherlands | Caucasians | KOOS | A | D | 56 | 20 | 36 | NR | NR | 9/11 | 13/23 |
| Krych AJ | 2017 | America | Caucasians | Lysholm score | B | C | 240 | 57 | 183 | 43 | 49 | 41/16 | 83/100 |
| Marsh JD | 2016 | Canada | Caucasians | WOMAC | A | E | 168 | 80 | 88 | 60.5 ± 9.9 | 58.3 ± 9.8 | 23/57 | 34/54 |
| Tuncay I | 2015 | Turkey | Caucasians | HSS | B | C | 146 | 52 | 94 | 51.7 (42-55) | 58.7 (45-69) | 42/10 | 79/15 |
| Yim JH | 2013 | Korea | Asians | Lysholm score | B | C | 108 | 58 | 50 | 58.3 ± 5.4 | 60.3 ± 4.5 | 7/51 | 2/48 |
| Takeuchi R | 2010 | Japan | Asians | KSS | B | C | 42 | 24 | 18 | 67 ± 7 | 77 ± 4 | 6/18 | 4/14 |
| Borjesson M | 2005 | Sweden | Caucasians | BOA score | B | C | 40 | 18 | 22 | 63 (4) | 63 (4) | 10/8 | 11/11 |
| Stukenborg-Colsman C | 2001 | Germany | Caucasians | KSS | B | C | 60 | 32 | 28 | 67 (60-79) | 67 (60-80) | 13/19 | 22/6 |
| Newman JH | 1998 | England | Caucasians | BOA score | C | D | 94 | 45 | 49 | 69.6 (53-89) | 69.8 (47-85) | 17/28 | 21/28 |

Notes: T = treatment; M = male; F = female; KOOS = knee injury and osteoarthritis outcome score; WOMAC = Western Ontario and McMaster Universities Osteoarthritis index; HSS = hospital for special surgery knee score; KSS = American knee society knee score; BOA = British Orthopaedic Association; A = non-operative treatment; B = osteotomy; C = unicompartmental knee arthroplasty; D = total knee arthroplasty; E = arthroscopic surgery.

**Supplementary Table 2. SMD and its 95%CI of functional scores of five treatments for KOA**

| **SMD (95%CI)** | | | | |
| --- | --- | --- | --- | --- |
| **A** | 0.98 (-0.52, 2.49) | 1.1 (-0.34, 2.54) | 0.51 (-0.54, 1.56) | 0.26 (-0.68, 1.2) |
| -0.98 (-2.49, 0.52) | **B** | 0.11 (-0.3, 0.53) | -0.47 (-1.55, 0.6) | -0.72 (-2.5, 1.05) |
| -1.1 (-2.54, 0.34) | -0.11 (-0.53, 0.3) | **C** | -0.59 (-1.57, 0.4) | -0.84 (-2.56, 0.89) |
| -0.51 (-1.56, 0.54) | 0.47 (-0.6, 1.55) | 0.59 (-0.4, 1.57) | **D** | -0.25 (-1.66, 1.16) |
| -0.26 (-1.2, 0.68) | 0.72 (-1.05, 2.5) | 0.84 (-0.89, 2.56) | 0.25 (-1.16, 1.66) | **E** |

Notes: SMD = standard mean difference; 95%CI = 95% confidence intervals; KOA = knee osteoarthritis; A = non-surgical treatment; B = osteotomy; C = unicompartmental knee arthroplasty; D = total knee arthroplasty; E = arthroscopic surgery.
